# ISG15 Is a Key Multidirectional Regulator of Cytosolic RNA- and DNA-Sensing Pathways and Type I Interferon Feedback Loops

**DOI:** 10.3390/ijms27198837

**Published:** 2026-10-03

**Authors:** Maleana G. White, Sergey Iordanskiy

**Affiliations:** 1 Department of Pharmacology & Molecular Therapeutics, Uniformed Services University of the Health Sciences, Bethesda, MD 20814, USA; maleana.white.ctr@usuhs.edu; 2 Henry M. Jackson Foundation for the Advancement of Military Medicine, Inc., Bethesda, MD 20817, USA; 3 Armed Forces Radiobiology Research Institute, Uniformed Services University of the Health Sciences, Bethesda, MD 20814, USA

**Keywords:** ISG15, ISGylation, UBL, IFN, PRR, MDA5, RIG-I, cGAS, STING, USP18

## Abstract

Activation of cytosolic RNA and DNA sensing pathways that normally recognize viral nucleic acids triggers the expression and release of type I interferons (IFN-I). This release drives the expression of interferon-stimulated genes (ISGs) that activate acute inflammation and counteract viral replication at various stages. Recent studies have highlighted how interferon-stimulated gene 15 (ISG15), a small, ubiquitin-like modifier, regulates cytosolic RNA and DNA sensing pathway proteins to control the antiviral response and inflammation. ISG15 is a multifunctional protein that acts as a secreted cytokine, binds freely to intracellular proteins, and covalently modifies proteins in a reversible process termed ISGylation. A dual regulatory molecule, ISG15 can both stabilize and destabilize proteins that promote and inhibit IFN-I signaling. Conversely, viruses have evolved strategies to disrupt and exploit ISG15-dependent antiviral responses to evade the host immune system. In this review, we examine how ISG15 regulates cytosolic RNA and DNA sensing pathway proteins to control IFN-I signaling and the antiviral response, highlighting it as a master regulator that fine-tunes IFN-I signaling and feedback. Recognizing the multifaceted action of ISG15 is essential for designing balanced therapeutic strategies across antiviral drug development, cancer immunotherapy, and chronic inflammatory disease management.

## 1. Introduction

While the innate immune system combines complex mechanisms of cellular defenses, such as phagocytosis, neutrophil extracellular traps, major histocompatibility complexes, and the complement system, inflammation represents a core immediate process, serving as the body’s first defense against infection or injury by initiating a rapid, non-specific response to destroy pathogens and signal for help. Uncontrolled inflammation also underlies many diseases and pathological processes including fibrosis. Therefore, mechanisms that regulate the immune response to prevent uncontrolled inflammation are critical for longevity and survival.

Pathogen-associated molecular patterns (PAMPs) and damage-associated molecular patterns (DAMPs) are detected by pattern recognition receptors (PRRs) that trigger rapid, systemic immune responses. Type I interferons (IFN-Is), including interferon alpha (IFN-α), IFN-β, IFN-ε, IFN-κ, and IFN-ω, are a major family of signaling proteins, or cytokines, that coordinate the body’s immediate immune response to viral infections and other dangers, including intracellular DAMPs [1,2,3,4]. Engagement of PRRs by PAMPs (e.g., viral DNA, RNA, and RNA:DNA duplexes) and DAMPs (e.g., mitochondrial RNA, and chromosomal and mitochondrial DNA fragments) initiates intracellular signaling cascades that facilitate phosphorylation and activation of transcription factors (TFs), including interferon regulatory factors (IRFs) and nuclear factor kappa-light-chain-enhancer of activated B cells (NF-κB), which orchestrate the synthesis and secretion of IFN-Is alongside a broad spectrum of pro- and anti-inflammatory cytokines and chemokines [2,5,6]. Secreted IFN-Is then bind to their receptors to stimulate synthesis of hundreds of interferon-stimulated genes (ISGs) that drive intracellular changes to eliminate infected and damaged cells [2,7,8]. While IFN-I signaling is critical in the early detection of infection and damage, continued expression and secretion of these interferons and pro-inflammatory cytokines can decrease cell proliferation and viability and give rise to a cytokine storm. Therefore, mechanisms that control this inflammatory response are critical to prevent hyper- and chronic inflammation.

An essential mechanism that regulates inflammatory signaling is the reversible modification of PRRs and associated downstream proteins with ubiquitin-like protein (UBL) modifiers [2,5,9,10,11]. A potent antiviral UBL is interferon-stimulated gene 15 (ISG15), a 15-kDa protein with both intracellular and extracellular functions. Intracellularly, ISG15 covalently modifies host and viral proteins in a reversible process termed ISGylation. While ISGylation of viral proteins impairs their function, interferes with their proper localization, and blocks the assembly and oligomerization of viral complexes [12], in the case of cellular targets, ISGylation generally stabilizes them to enhance the host cell antiviral response [12,13,14,15,16,17,18]. Intracellular ISG15 can also non-covalently bind to proteins, including ubiquitin-specific peptidase 18 (USP18), a negative regulator of IFN-I signaling [19]. USP18 in coordination with free intracellular ISG15 acts as a specialized cysteine protease that reverses ISGylation of host target proteins to turn off IFN-I signaling and prevent dangerous hyper-inflammation [20]. Extracellularly, ISG15 functions as a cytokine. Upon secretion, ISG15 binds to cell-surface integrin receptors such as lymphocyte function-associated antigen 1 (LFA-1) on natural killer (NK) and T cells to robustly induce IFN-II (IFN-γ) secretion [21].

While ISG15 generally promotes IFN-I signaling and the antiviral response in virus-infected cells, it can also trigger damaging inflammation and pathological immune system dysfunction [22,23,24,25]. In this review, we synthesize insights into the ISG15-dependent regulation of a specific subset of PRR pathways that trigger IFN-I expression: cytosolic nucleic acid-recognizing PRR pathways. We focus on ISGylation in response to viral infection and ISG15-USP18-mediated feedback regulation to turn off IFN-I signaling. We also cover findings about viral mechanisms that antagonize ISG15-dependent activation of cytosolic PRR pathways to subvert the host immune response.

## 2. Cytosolic Nucleic Acid-Sensing PRR Pathways Activate IFN-I Signaling

### 2.1. Cytosolic RNA- and DNA-Sensing PRR Pathway Components

Cytosolic nucleic acid-sensing PRRs are grouped into two main types: RNA and DNA sensors. These receptors perceive both foreign and endogenous viral nucleic acids to trigger the synthesis of IFN-Is, which then stimulate the expression of ISGs, including viral restriction factors and numerous cytokines and chemokines.

**Figure 1:**
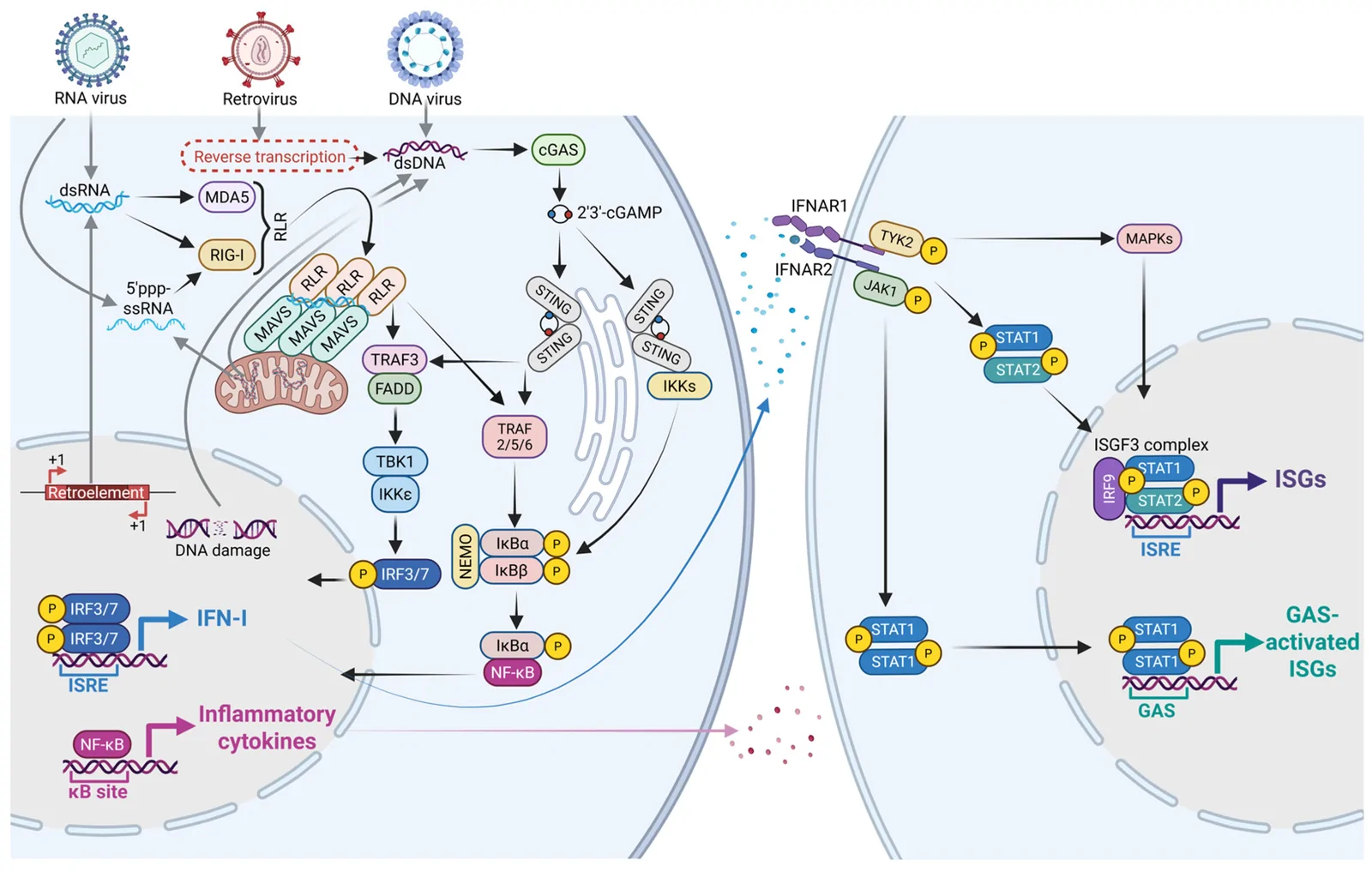
Overview of cytosolic nucleic acid-sensing pattern recognition receptor pathways that stimulate synthesis of type I interferons (IFN-Is) and interferon-stimulated genes (ISGs). Cytosolic double-stranded DNA (dsDNA) or RNA:DNA hybrids derived from viral infection or damaged DNA binds to cyclic GMP-AMP synthase (cGAS), triggering the production of 2′,3′-cGAMP. This small molecule then activates the stimulator of interferon genes (STING) adapter protein, which oligomerizes on the surface of intracellular membranes including the endoplasmic reticulum and Golgi apparatus. Double-stranded RNA (dsRNA), in addition to viral 5′ppp-ssRNA, from infection, mitochondrial damage, or retroelement transcription is recognized by retinoic acid inducible gene I (RIG-I), while melanoma differentiation-associated protein 5 (MDA5) recognizes only dsRNA, driving the oligomerization of these receptors and subsequent interaction with mitochondrial antiviral signaling protein (MAVS) on the outer mitochondrial membrane. Both STING and MAVS activate two downstream signaling cascades: (1) phosphorylation, dimerization, and nuclear translocation of interferon response factors 3/7 (IRF3/7), which bind to interferon-stimulated response elements (ISREs) in the promoters of IFN-I genes; and (2) dimerization and nuclear translocation of NF-κB to drive pro-inflammatory gene expression. Secreted IFN-Is bind autocrine and paracrine interferon-alpha/beta receptor subunits 1 and 2 (IFNAR1/2), initiating a Janus kinase 1-signal transducer and activator of transcription (JAK1-STAT) cascade that phosphorylates and dimerizes STAT1 and STAT2. This heterodimer recruits IRF9 to form the interferon-stimulated gene factor 3 (ISGF3) complex, which binds to ISREs to induce the expression of ISGs. Gray arrows indicate nucleic acids sources, while black arrows indicate signaling pathways. Created in BioRender. White, M. and Iordanskiy, S. (2026) https://BioRender.com/t25pdl8.

The most abundant cytosolic RNA sensors belong to the DExD/H-box RNA helicase family: melanoma differentiation-associated protein 5 (MDA5), which binds to long (>2000 bp) double-stranded RNA (dsRNA) [26], and retinoic acid-inducible gene I (RIG-I), which binds to short dsRNA and single-stranded RNA (ssRNA) containing blunt 5′-triphosphate (5′ppp) or 5′-diphosphate groups [27,28,29]. Together with laboratory of genetics and physiology 2 (LGP2), these proteins constitute the RIG-I-like receptors, or RLRs [30]. Upon RNA binding and oligomerization through both RNA- and polyubiquitin-dependent mechanisms [31], these RLRs interact with the adaptor protein mitochondrial antiviral signaling protein (MAVS) on the outer mitochondrial membrane. MAVS then stimulates TANK-binding kinase 1 (TBK1) and IκB kinase-ε (IKKε) to phosphorylate and activate IRF3/7 and NF-κB, respectively. While IRF3/7 predominantly induce transcription of IFN-Is, NF-κB regulates the expression of numerous other pro-inflammatory cytokines and chemokines [30,32,33] (Figure 1).

The most abundant cytosolic DNA sensor is cyclic GMP-AMP synthase (cGAS), which primarily recognizes dsDNA and, under certain conditions, RNA:DNA hybrids such as reverse-transcription intermediates and R-loops. Other PRRs capable of detecting dsDNA include Absent In Melanoma 2 (AIM2) inflammasome, interferon gamma-inducible protein 16 (IFI16), and DEAD-box helicase 41 (DDX41) [34,35]. Upon binding to dsDNA or RNA:DNA hybrids, cGAS dimers synthesize 2′,3′-cGAMP from ATP and GTP. The 2′,3′-cGAMP molecules then activate the adaptor protein stimulator of interferon genes (STING), which is primarily localized to the endoplasmic reticulum (ER) membrane [36,37].

The MAVS- and STING-mediated pathways share the same downstream signaling components that induce IRF3/7- and NF-κB-dependent gene expression. Secreted IFN-Is bind to the interferon-alpha/beta receptor (IFNAR) to activate synthesis of ISGs through Janus kinase 1 (JAK1)-mediated phosphorylation of STAT1 and STAT2 TFs. Phospho-STAT1 and phospho-STAT2 dimerize before recruiting IRF9 and forming the interferon-stimulated gene factor 3 (ISGF3) complex, which translocates into the nucleus to activate transcription of numerous ISGs whose promoters contain interferon-stimulated response elements (ISREs) [2,7,8] (Figure 1). Proteins encoded by ISGs exhibit diverse functions spanning enzymatic, regulatory, and signal transducing roles to inhibit viruses at various stages of their replication cycle and regulate IFN-I signaling through positive and negative feedback loops.

### 2.2. Direct and Indirect Crosstalk Between Cytosolic RNA and DNA Sensing Pathways

The RNA- and DNA-sensing cytosolic PRR pathways are directly and indirectly intertwined. At the functional level, RIG-I signaling strongly stimulates expression of STING, indicating indirect crosstalk between these pathways [38,39,40]. Moreover, both RIG-I and STING were found to be involved in the DNA damage repair response. RIG-I was demonstrated to be recruited to the site of double-stranded breaks (DSBs) in chromosomal DNA, where it suppressed repair and increased sensitivity to ionizing-radiation-induced DNA damage but reduced efficiency of retroviral cDNA integration, suppressing the non-homologous end-joining pathway [41]. Similarly, accumulation of R-loops, the intermediate structures of transcription that consist of RNA:DNA hybrids and ssDNA, due to DSBs [42] or a DDX41 deficiency, triggered cGAS/STING-mediated inflammation in non-differentiated hematopoietic stem cells [43]. Conversely, at the molecular level, RNA viruses, such as dengue virus and hepatitis C virus (HCV), express papain-like proteases (PLpro) that either directly cleave STING or disrupt its interaction with the RIG-I-MAVS complex [44,45].

In addition to converging on DNA repair pathways, RNA and DNA sensors overlap in their response to retroelements. Retroelements make up approximately 42% of the human genome, with a subset of virus-like elements—endogenous retroviruses (ERVs)—comprising 8.3% of the genome. Retroelements including ERVs can be used as templates to synthesize dsRNA due to transcription occurring in both the sense and antisense directions or RNA:DNA duplexes due to the activity of endogenous reverse transcriptase [46]. Chiappinelli and colleagues demonstrated that treatment of multiple human epithelial ovarian cancer cell lines with the DNA methyltransferase inhibitor 5-azacytidine (Aza) upregulated ERV genes that were typically hypermethylated in these cells, triggering activation of the IFN-I response and apoptotic pathways [47]. This study also revealed that in a mouse melanoma model, combinatorial treatment with Aza and the immune checkpoint therapy anti-CTLA4 increased the responsiveness of the mice to the therapy, suggesting that Aza could bolster the anti-tumor effects of anti-CTLA4 [47]. Later, Min and colleagues found that ERV levels increased in response to exposure of skin cells to UV radiation, and these levels correlated with activation of RLR pathway components [48]. Work done by our group has revealed that exposure of human monocytes and macrophages to ionizing radiation increased expression of ERVs and cytosolic DNA and RNA sensors in these cells, facilitating the activation of IFN-I and NF-κB signaling pathways [46,49,50]. Despite the many ways in which these cytosolic sensors are triggered, regulatory mechanisms are necessary to fine-tune their immune response according to PAMP/DAMP levels.

## 3. ISG15 Levels Are Regulated by IFN-I Signaling

### 3.1. ISG15 Protein Structure and E1/E2/E3-Mediated Conjugation

ISG15 exists in conjugated and unconjugated forms, with the free form functioning both intra- and extracellularly. During ISGylation, a covalent bond is formed between glycine (G) residues in the C-terminal LRLRGG motif of ISG15 and lysine (K) residues in host and viral target proteins; the ability of ISG15 to be covalently conjugated is lost if these glycine residues are mutated to alanine [51]. Similar to ubiquitination, ISGylation is catalyzed in three enzymatic steps beginning with the transfer of ISG15 to an E1 ubiquitin-activating enzyme, which exposes the LRLRGG motif before transferring ISG15 to a highly reactive cysteine residue in an E2 ubiquitin-conjugating enzyme. An E3 ubiquitin ligase transfers ISG15 to a target protein in the final step of the reaction. The major enzymes catalyzing each of these steps have been identified in multiple species. In humans and mice, ubiquitin-like modifier-activating enzyme 7 (UBA7)/ubiquitin-activating enzyme E1-like (UBE1L) functions as the primary E1 ubiquitin-activating enzyme, while ubiquitin-conjugating enzyme human 8 (UBCH8) and ubiquitin-conjugating enzyme mouse 8 (UBCM8) are, to date, the only identified E2 enzymes catalyzing ISGylation in humans and mice, respectively. Multiple E3 ubiquitin ligases are involved in ISGylation, with HECT and RLD domain-containing E3 ubiquitin protein ligase 5 (HERC5) and HERC6 functioning as the primary modulators in humans and mice, respectively. It was also demonstrated that UbcH8~ISG15 selectively reacts with the E6-AP carboxyl terminus (HECT) and RING-between-RING (RBR) families of E3 ubiquitin ligases but not the interesting new gene (RING) family; among the HECT family, HERC5 is the main E3 ubiquitin ligase that transfers ISG15 to other proteins [52].

Tripartite motif-containing protein 25 (TRIM25), a member of the HECT protein family, and Ariadne RBR E3 ubiquitin protein ligase 1 (ARIH1) are additional E3 ubiquitin ligases that ISGylate protein substrates. TRIM25 is critical for K63-linked ubiquitination and activation of RIG-I, and while it is not the predominant E3 ubiquitin ligase controlling ISGylation, evidence suggests that it is, indeed, involved in this process [53,54,55]. Notably, RIG-I promotes ISGylation by regulating TRIM25 mRNA and protein levels. Treatment of an acute promyelocytic leukemia human cell line, NB4, with all-trans retinoic acid, a potent inducer of RIG-I, and treatment of a RIG-I-inducible acute monocytic leukemia human cell line, U937-RIG-I, with doxycycline to express RIG-I, increased TRIM25 transcript and protein levels in addition to total protein ISGylation [55]. Consistently, inhibition of TRIM25 and ISG15 translation with short hairpin RNA in these cell lines with elevated RIG-I expression decreased ISGylation, suggesting that RIG-I-induced ISGylation requires TRIM25 [55].

### 3.2. Free Intracellular ISG15 and Total Protein ISGylation Are Increased by Stimuli That Activate Cytosolic RNA and DNA Sensing Pathways

The activation of MAVS- and STING-mediated signaling pathways that drive IFN-I signaling has been shown to increase abundance of both free intracellular ISG15 and global ISGylation of host and viral proteins. ISGylation is widely studied in the context of viral infection, with nucleic acids from both RNA viruses—such as influenza A virus (IAV), influenza B virus (IBV), Ebola, human immunodeficiency virus type 1 (HIV-1), Middle East respiratory syndrome coronavirus (MERS-CoV), severe acute respiratory syndrome coronavirus 2 (SARS-CoV-2), and foot-and-mouth disease virus (FMDV)—and DNA viruses—such as human cytomegalovirus (CMV), Kaposi sarcoma herpesvirus, and vaccinia virus—stimulating cytosolic RNA and DNA sensors to drive accumulation of free ISG15 and ISGylated proteins [56,57,58]. However, bacterial infections can also trigger a similar response. Accordingly, experiments demonstrated this phenomenon in human THP-1 monocytes that were differentiated to macrophages and subsequently infected with *Mycobacterium smegmatis* (*M. smegmatis*). Infection of THP-1 cells with *M. smegmatis* overexpressing EsxN, a secreted effector protein and virulence factor, reduced transcript levels of potent pro-inflammatory cytokines—tumor necrosis factor alpha (TNF-α), IL-6, IL-12, and IL-18—as well as the NLR family pyrin domain-containing 3 (NLRP3) inflammasome [59]. At the same time, EsxN overexpression boosted cGAS–STING pathway activation, enhanced total protein ISGylation, and increased bacterial survivability in host cells, revealing a mechanism whereby *M. smegmatis* evades host immune responses through hijacking the cGAS-STING-ISG15 signaling axis to increase ISGylation of beneficial substrates [59]. In another study utilizing human primary renal proximal tubule epithelial cells, Tachizaki and colleagues demonstrated that treatment with synthetic dsRNA poly(I:C) upregulated transcription of RIG-I, ISG15, UBA7/UBE1L, and USP18 in a time- and dose-dependent manner, with increased free intracellular ISG15 and total protein ISGylation also observed [60]. Crucially, while knockdown of the E1 ubiquitin-activating enzyme UBA7/UBE1L diminished total protein ISGylation, silencing the deISGylating enzyme USP18 significantly enhanced it [60]. These opposing effects eloquently illustrate a molecular balancing act to keep cellular ISGylation and, consequently, IFN-I signaling in check.

Accumulation of cytoplasmic dsDNA due to DNA damage (from aberrant replication, for instance) also engages the cGAS-STING pathway to alter accumulation of free intracellular ISG15 and ISGylated proteins. When Nijmegen breakage syndrome 1 (Nbs1) and meiotic recombination 11, proteins involved in the dsDNA break repair response, were deleted from mouse embryonic fibroblasts (MEFs), increased accumulation of free intracellular ISG15 and ISGylated proteins was observed [61]. This accumulation required functional cGAS-STING-TBK1 signaling, as chemical inhibition of TBK1 with MRT67307 or genetic knockout of cGAS blocked the accumulation of free intracellular ISG15 and ISGylated proteins in Nbs1-deficient MEFs [61]. Moreover, MEFs that were deficient in ISG15 contained elevated numbers of micronuclei indicative of improper DNA replication [61]. Consistent with these observations, ISGylation of proteins that accumulate at the site of DNA replication forks was observed, suggesting that ISGylation is important in facilitating DNA replication to prevent damage from occurring during this process [61]. Nevertheless, the effects of free intracellular ISG15 on DNA replication and the dsDNA break repair response under these conditions are still unclear. In another study, treatment of human fibrosarcoma HT1080 cells with the *Salmonella enterica* (*S. enterica*) typhoid toxin to increase host DNA damage resulted in elevated levels of free intracellular ISG15 and ISGylated proteins, reduced *S. enterica* presence in host cells, and reduced apoptosis of host cells [62]. When STING and TBK1 were knocked down with targeted siRNA in these cells, the increase in free intracellular ISG15 levels following toxin treatment was lost, suggesting a role of free ISG15 in mitigating host DNA damage and *S. enterica* infectivity [62]. However, the effect on total protein ISGylation was not examined in these STING and TBK1 knockdown cells, so the link between STING-TBK1-ISGylation under these conditions remains unclear.

While expression of ISG15 is strongly dependent on IFN-I signaling, there is evidence that accumulation of free intracellular ISG15 can be regulated independently of IFN-I signaling, as blocking of IFNAR in HeLA cells with the chemical inhibitor B18R or genetically modified U5A cells lacking the IFNAR2 subunit, followed by infection of these cells with *Listeria monocytogenes* (*L. monocytogenes*), resulted in increased abundance of free ISG15 [63]. However, the authors of this study did not examine total protein ISGylation in response to inhibition of IFNAR-mediated signaling under these conditions, so whether ISGylation can be controlled by IFN-I-independent mechanisms under these conditions remains unclear.

Taken together, these findings reveal an important role for the activation of nucleic acid-sensing PRR pathways in increasing free intracellular ISG15 and total protein ISGylation in the context of pathogen attack, DNA replication, and DNA damage repair, although the exact contributions of free intracellular ISG15 versus ISGylation in these responses still require further untangling.

### 3.3. Viral Restriction Activity of ISG15

The antiviral roles of ISG15 have been reviewed previously [24,56,64], with new work still rapidly emerging. One mechanism by which ISG15 thwarts viral attack is through the direct ISGylation of viral proteins. The E3 ubiquitin ligases HERC5, TRIM25, and ARIH1 catalyze the transfer of ISG15 onto viral proteins. HERC5 associates with polyribosomes. As demonstrated in HEK293T cells, HERC5 co-translationally tethers ISG15 onto viral proteins [65]. ISGylation of nascent viral proteins blocks their ability to fold and, subsequently, decreases viral infectivity, representing an important defense mechanism against viruses such as HIV-1 and SARS-CoV-2 [66]. Moreover, HERC5 was shown to block the replication of SARS-CoV-2 through ISGylation of non-structural protein 8 [66] as well as proteins involved in viral RNA synthesis [67]. To evaluate other SARS-CoV-2 viral protein targets of ISGylation, FLAG-tagged SARS-CoV-2 nucleocapsid (N), envelope, and membrane proteins were co-expressed with the ISGylation machinery in HEK293T cells [67]. Co-immunoprecipitation revealed that ISG15 directly bound to and modified the N protein only, demonstrating that SARS-CoV-2 N protein is selectively ISGylated [67]. ISGylation of this viral protein blocked oligomerization and subsequent viral assembly [67]. In an immortalized fish cell line, epithelioma papulosum cyprini (EPC), overexpression of zebrafish ISG15 increased levels of ISGylated viral proteins upon infection with various fish RNA and DNA viruses, and this outcome was further exacerbated by co-expression of ISG15 with plasmids expressing nonviral or viral phosphoproteins belonging to infectious hematopoietic necrosis virus [68].

ISG15 can also indirectly perturb viral attacks through the ISGylation of host proteins. In HeLA cells and pluripotent stem-cell-derived macrophages and endothelial cells, ISGylation of proteins involved in glycolysis facilitates metabolic reprogramming during an immune response, a process that can be reversed by viral deISGylation mechanisms to meet the energy demands of viral replication [24,69,70]. Furthermore, ISGylation of cytosolic RNA and DNA sensors generally stabilizes these proteins to bolster IFN-I signaling, a mechanism that is discussed in more detail in Section 4.

Free intracellular and extracellular ISG15 regulates innate and adaptive immunity in response to viral infection and in cancerous cells. Free intracellular ISG15 was shown to restrict budding of retroviruses by preventing the viral Gag protein from interacting with components of the endosomal sorting complex required for transport (ESCRT) machinery in HEK293T cells [71]. In cultures of primary human umbilical vein endothelial cells and in mouse female genital tract organoids, extracellular ISG15 was found to bind to interleukin-18 receptor alpha (IL-18Rα) to stimulate the production and secretion of IFN-ɣ in response to *Chlamydia trachomatis* infection [72]. A non-ISGylation role for ISG15 in the antiviral response was also observed in mice: In C57BL/6 neonatal mice infected with Chikungunya virus, viral load decreased in response to poly(I:C) treatment, an effect that was lost in mice lacking ISG15 but not UBA7/UBE1L, suggesting that, in this specific situation, ISG15 decreased viral load not through ISGylation but through a different IFN-I-dependent signaling mechanism [73]. In the context of cancer, a cell-based assay using NK-92 cells, which are immortalized human NK cells derived from a patient with non-Hodgkin’s lymphoma, revealed that free extracellular ISG15 interacted with the CD11α chain of receptor LFA-1 to promote cytokine secretion [21]. In addition to NK, extracellular ISG15 can also activate cytotoxic CD8+ T cells to promote adaptive immunity [74].

Together, these findings highlight the various mechanisms that ISG15 uses to control innate and adaptive immunity in response to viral infection and in pathological contexts such as cancer. In the next section, we explore the regulatory feedback mechanisms of ISG15 on IFN-I signaling through ISGylation of cytosolic RNA and DNA sensors and non-covalent interactions with RIG-I and USP18.

## 4. ISG15 Regulates Cytosolic RNA- and DNA-Sensing Pathways to Control IFN-I Signaling

### 4.1. Identification of Targets of ISGylation

Immunoprecipitation (IP) of ISG15 or peptides containing diglycine tags, which remain after digestion of ISG15 from substrates, coupled with treatments that induce or perturb expression of ISG15 or ISGylation machinery, have identified hundreds of targets of ISGylation, with several of these landmark studies previously reviewed [75]. Notably, two studies published in 2005 and 2006 already made it possible to identify a broad landscape of ISG15 substrates. In the first study, extraction of proteins from IFN-β-treated HeLa cells harboring double-affinity tagged ISG15 coupled with mass spectrometry (MS) identified 158 targets of ISGylation and validated several key antiviral proteins, including RIG-I, Myxovirus resistance 1 (MX1)/Myxovirus resistance protein A (MxA), interferon-induced protein with tetratricopeptide repeats 1 (IFIT1), and several others [76]. In the second study, MEFs, promonocytic U937 cells, and murine bone marrow-derived macrophages (BMMs) were treated with IFN-α or IFN-β [77]. IP of ISG15 followed by MS analysis revealed 21 total targets of ISGylation in these cells spanning a wide array of biological functions [77]. In another study using HeLa cells transfected with ISGylation machinery (UBA7/UBE1L, UBCH8/UBE2L6, and HERC5), 10 targets were identified [78]. More recently, treatment of porcine alveolar macrophages (PAMs) with IFN-α then IP of ISG15 with a monoclonal antibody followed by MS analysis identified 98 targets and 190 ISGylation sites, with many of these targets having more than one ISGylation site [79]. Studies that employed diglycine proteomics identified larger sets of targets of ISGylation. In one study, proteins from the livers of *L. monocytogenes*-infected wild-type and *ISG15*-deficient mice were trypsin-digested and then immunoprecipitated with an antibody targeted to diglycine tags [80]. This study identified 434 bona fide targets of ISGylation and 930 modification sites [80]. To identify the targets of human HERC5- or murine HERC6-mediated ISGylation, Zhao and colleagues knocked out HERC5 in HERK293T cells then reconstituted these cells with ISGylation machinery and HERC5 or HERC6 [81]. Using an antibody that specifically identified lysine-linked diglycine tags in digested proteins from these cells, over 2700 total protein targets and over 7000 modification sites were identified [81]. These studies provide insight into the proteins and pathways regulated by ISG15.

### 4.2. ISGylation of Cytosolic RNA and DNA Sensors and Adaptors

Available data indicate that while ISG15 covalently binds to diverse nuclear, cytosolic, and transmembrane substrates, its primary targets are central to innate immune responses against viral and bacterial infections, as well as the maintenance of genomic stability, where ISGylation functions as a surveillance mechanism during DNA replication. Generally, ISGylation acts as a stabilizing post-translational modification (PTM) that enhances the functional activity of these critical proteins. The experimentally validated activities of ISG15, including ISGylation and non-covalent ISG15-protein interactions for the major cytosolic DNA and RNA sensors and the adaptor protein STING are summarized in Table 1. Currently, there is no evidence that the adaptor protein MAVS is regulated by ISGylation. In the remaining sections, we review how ISGylation of cytosolic PRR sensors and adaptors alters IFN-I signaling and how host and viral processes modulate ISG15-dependent regulation of these pathways.

#### 4.2.1. MDA5

**Figure 2:**
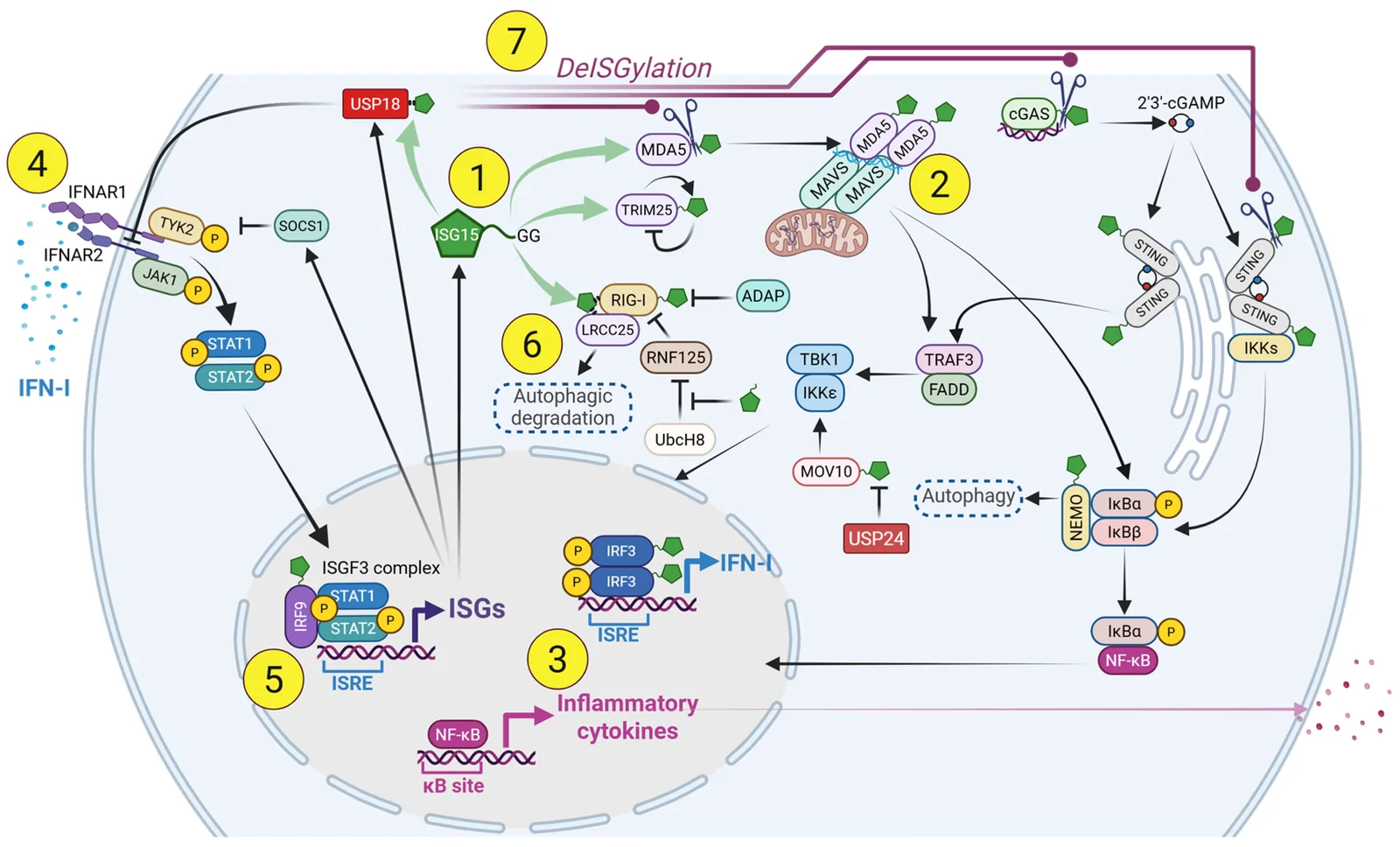
Multifaceted regulation of cytosolic nucleic acid sensing and type I interferon (IFN-I) signaling by interferon-stimulated gene 15 (ISG15). IFN-I signaling induces the expression of ISG15, which covalently modifies key mediators of innate immune pathways in a reversible process termed ISGylation (1). Positive regulation of nucleic acid sensing pathways. ISGylation of cytosolic sensors and adapters enhances downstream signaling: ISGylation of melanoma differentiation-associated protein 5 (MDA5) promotes its oligomerization and binding to mitochondrial antiviral signaling protein (MAVS) to amplify dsRNA sensing. ISGylation of the RNA helicase Moloney leukemia virus 10 (MOV10) boosts TANK-binding kinase 1 (TBK1)/IκB kinase epsilon (IKKε) activity and interferon response factor 3 (IRF3)-dependent IFN-I expression; and ISGylation of cyclic GMP-AMP synthase (cGAS)/stimulator of interferon genes (STING) enhances cytosolic double-stranded DNA (dsDNA)-driven IFN-I expression. Additionally, ISGylation of NF-κB essential modulator (NEMO) within the IκBα/β complex facilitates NF-κB-dependent transcription of pro-inflammatory genes (2, 3). Secreted IFN-Is bind to IFNAR (4) and induce ISG expression through the binding of the interferon-stimulated gene factor 3 (ISGF3) complex to interferon-stimulated response elements (ISREs); ISGylation of the interferon response factor 9 (IRF9) protein within this complex enhances its transcriptional activation function (5). Negative regulation of sensors: Conversely, ISGylation of retinoic acid-inducible gene I (RIG-I) targets it for proteasomal degradation, blunting its downstream signaling cascade and therefore IFN-I expression (6). Transcriptional and negative feedback modulation: Within the ISGF3 complex, ISGylation of IRF9 enhances transcription of downstream ISGs, including the major deISGylase ubiquitin specific peptidase 18 (USP18). USP18, non-covalently bound to ISG15, subsequently suppresses IFN-I signaling by blocking the IFNAR2 subunit and deISGylating cGAS, STING, and MDA5, which destabilizes these proteins and turns off IFN-I expression (7). Purple lines indicate the deISGylation of protein substrates; dotted lines shows non-covalent binding with free intracellular ISG15. Created in BioRender. White, M. and Iordanskiy, S. (2026) https://BioRender.com/t25pdl8.

MDA5 is ISGylated to promote its oligomerization and subsequent activation of the IFN-I pathway. Liu and colleagues demonstrated that ISGylation of murine MDA5, but not RIG-I, was required for IFN-I signaling in MEFs, as MDA5- and RIG-I-dependent increases in IFN-β secretion were lost only in FLAG-MDA5-transfected cells when ISG15 was knocked out, while FLAG-RIG-I-transfected cells continued to secrete IFN-β despite the absence of ISG15 [82]. This group also found that ISGylation of MDA5 at K23 and K43 was required for the multimerization of this protein, a process critical for activating synthesis of IFN-Is and ISGs in response to viral infection [82]. Similarly, primary mouse dermal fibroblasts from mice with mutated MDA5 harboring mutations at residues K23 or K43 had lower levels of MDA5 oligomerization [99]. These mice also had a lower capacity to elicit a IFN-I response after exposure to SARS-CoV-2 RNA and after infection with West Nile virus, IAV, and encephalomyocarditis virus (EMCV), while also exhibiting elevated viral titers, revealing the importance of MDA5 ISGylation and subsequent oligomerization in stimulating the antiviral response to block viral replication in cells [99]. ISGylation of MDA5 in the context of IFN-I signaling is illustrated in Figure 2.

#### 4.2.2. RIG-I

The role of ISG15 and ISGylation of RIG-I in its ability to activate IFN-I signaling is highly complex (Figure 2). The primary PTM regulating RIG-I is ubiquitination, with K48-linked ubiquitination leading to proteasomal degradation of RIG-I and, therefore, negative regulation of downstream IFN-I signaling, and K63-linked ubiquitination leading to RIG-I activation and bolstering the antiviral response [100,101,102]. Nevertheless, several studies revealed that direct ISGylation of RIG-I occurs in cells grown in culture, with data limited to human HeLa, HEK293T, HepG2, African green monkey COS-7, and murine RAW264.7 cells, as well as in immortalized bone marrow-derived macrophages (iBMMs) from mice [76,83,84,85]. In the study performed by Zhang and colleagues, which examined ISGylation sites in livers from *L. monocytogenes*-infected mice, two ISGylation sites at K257 and K456 on RIG-I were identified [80]. Furthermore, two of the studies cited earlier examining global targets of ISGylation identified RIG-I as a target [76,80].

ISG15 functions in different regulatory pathways involved in the stabilization and degradation of RIG-I. In one study, the E3 ubiquitin ligase RING finger protein 125 (RNF125), which catalyzes the ubiquitin-mediated degradation of RIG-I, directly bound to UBCH8/UBE2L6, which prevented RNF125 from ubiquitinating RIG-I [84]. However, when ISG15 was ectopically expressed, UBCH8/UBE2L6 dissociated from RNF125 and associated with ISG15 instead, which freed RNF125 from its association with UbcH8/UBE26L, allowing it to ubiquitinate RIG-I [84]. On the other hand, ectopic expression of UBCH8/UBE2L6 suppressed RNF125’s ubiquitination activity and promoted ISGylation [84]. By analyzing this regulatory circuit, the authors established that UBCH8/UBE2L6 and ISG15 function as reciprocal regulators of RNF125 E3 ubiquitin ligase activity, ultimately controlling the balance between the degradation and stabilization of RIG-I [84]. Another study showed that non-covalent association between ISG15 and RIG-I promoted the degradation of RIG-I through the autophagosomal pathway to inhibit IFN-I signaling. Here, leucine-rich repeat-containing protein 25 (LRRC25) directly bound to RIG-I and recruited it to autophagic cargo receptor p62/sequestosome 1 for degradation [103]. The interaction between LRRC25 and RIG-I required ISG15, as COS-7 cells that lacked *ISG15* did not exhibit this interaction [103]. Furthermore, ISG15 with a mutated LRLRGG motif was still able to drive LRRC25-mediated autophagic degradation of RIG-I [103], revealing that ISGylation is not important in this process. In contrast, immortalized fallopian tube epithelial cells (FTE-194) that lacked *ISG15* or UBA7/UBE1L had lower protein levels of unconjugated RIG-I as well as lower IFN-I signaling, suggesting that ISGylation stabilized RIG-I in these cells [86]. However, whether this effect was a result of direct ISGylation of RIG-I or an indirect effect of the ISGylation of other proteins was not clear from this study.

There is also evidence that ISGylated RIG-I itself functions as a negative regulator of IFN-I signaling. In one study using COS-7 cells grown in culture, transfection of these cells with ISGylation machinery lowered levels of unconjugated RIG-I and increased levels of the ISGylated form, and yet these cells also had reduced ISRE promoter activity as determined by a luciferase assay [85]. Furthermore, MEFs that were deficient in UBA7/UBE1L had increased levels of unconjugated RIG-I as well as increased ISRE and positive regulatory domain III promoter activity [85]. ISGylated RIG-I functioned as a negative regulator of IFN-I signaling [85]. In another study, adhesion and degranulation-promoting adapter protein (ADAP) was found to block ISGylation of RIG-I by directly binding to it [83]. Immortalized BMMs that were deficient in *ADAP* had elevated levels of ISGylated RIG-I and a concomitant reduction in IFN-I signaling, which promoted Sendai virus (SeV) replication in these cells [83].

Perhaps the degradation of RIG-I can diminish or enhance IFN-I signaling, depending on whether the non-ISGylated or ISGylated form of RIG-I is degraded, respectively. Together, these studies point towards a complex role ISG15 in modulating IFN-I signaling through its regulation of RIG-I and reveal a strong dependence on cell type and stimulus. ISG15 regulation of RIG-I in the context of IFN-I signaling is illustrated in Figure 2.

#### 4.2.3. cGAS

cGAS is ISGylated by multiple E3 ubiquitin ligases to promote its oligomerization and subsequent activation of IFN-I signaling through the STING pathway. cGAS was found to be ISGylated at K21, K187, K219, and K458 by HERC5 [87]. Using immunofluorescence in conjunction with confocal microscopy, cGAS was revealed to colocalize with HERC5 and ISG15 in human aortic endothelial cells after transfection with the ISGylation machinery or after treatment with IFN-β [87]. Native-PAGE immunoblot revealed that HERC5-mediated ISGylation of cGAS was important for its oligomerization [87]. The physiological relevance of this pathway is highlighted in vivo, where mice deficient in ISG15 or HERC6 are significantly more vulnerable to herpes simplex virus 1 (HSV-1) infection [87]. Similarly, Arih1, another E3 ubiquitin ligase, was identified as a protein that directly interacts with cGAS, and mice lacking *ARIH1* had a reduced antiviral response to HSV-1 infection as indicated by a reduction in IFN-I at the RNA and protein levels [88]. It was revealed that ARIH1 catalyzed the mono-ISGylation of cGAS at K187, as MEFs lacking *cGAS* that were reconstituted with FLAG-tagged or untagged cGAS possessing a lysine-to-asparagine mutation at position 173 abolished ISGylation of cGAS after HSV-1 infection [88]. ISGylation of cGAS in the context of IFN-I signaling is illustrated in Figure 2.

#### 4.2.4. STING

STING undergoes ISGylation in response to viral infections to promote IFN-I signaling (Figure 2). In the presence of HIV-1 cDNA, ISGylation at residues K224, K236, K289, K347, K338, and K370 was detected, and this ISGylation was necessary for oligomerization of STING to activate IFN-I signaling [89]. Transfection of HEK293A cells and macrophage-like, differentiated THP-1 cells with wild-type STING or STING containing single mutations at various lysine residues revealed reduced ISGylation in the mutant cells, with the most pronounced effects in the K289 mutant [89]. These mutations resulted in decreased IFN-I production in HEK293A and THP-1 cells after HIV-1 infection [89]. Both cell lines expressing mutant STING had decreased dimerization and oligomerization of STING as determined by SDS-PAGE and native-PAGE, respectively [89]. Consistent with ISGylation of STING as an important driver of the innate immune response, Zhang and colleagues showed that siRNA-mediated knockdown of *ISG15* reduced levels of phospho-TBK1 and phospho-IRF3 in zebrafish [91]. Specifically, the authors generated a STING mutant in which all but one ISGylation lysine site was abolished, revealing that ISGylation of K221 and K276 had the highest levels of ISGylation [91]. ISGylation of these sites antagonized K48 ubiquitination-mediated degradation of STING and promoted its oligomerization and activation of IFN-I signaling [91].

Consistent with the above findings, an in vivo study demonstrated that murine HERC6 is important for STING-mediated IFN-I signaling [90]. *HERC6*-deficient mice had reduced levels of phosphorylated TBK1 and phosphorylated IRF3, as well as decreased mRNA and protein levels of IFN-β and TNF-α [90]. These mice had greater susceptibility to HSV-1 as revealed by higher levels of viral RNA in brain tissue [90]. HERC5 directly interacted with STING, as immunoprecipitation of HEK293T cells transfected with Myc-STING and FLAG-HERC5 or FLAG-HERC6 after pull-down of FLAG revealed increased levels of Myc compared to cells transfected with Myc-STING only [90]. This study revealed that ISGylation was critical for STING oligomerization [90]. This study also identified a novel STING ISGylation site at K150, with HERC5 being the predominant E3 ligase to ISGylate it, and demonstrated the function of SARS-CoV-2 in deISGylating STING [90]. Another study revealed that ISGylation of STING at K236, K338, K347, and K370 stabilized the protein by preventing its degradation through the autophagic pathway [104], thereby enhancing its downstream immunostimulatory function. This study also showed that USP18 deISGylated STING, a process that could be tempered by a compound (tanshinone IIA sulfonate) that blocks USP18 [104]. Activation of STING-mediated IFN-I signaling reduced tumor size and elevated NK cell-mediated targeting of these cells [104]. ISGylation of STING in the context of IFN-I signaling is illustrated in Figure 2.

#### 4.2.5. Other IFN-I Pathway Proteins

In addition to the proteins above, other proteins involved in the IFN-I signaling pathway, such as IRF3, IRF9, STAT1, MOV10, NEMO, TRIM25, and USP18, are regulated by ISG15, with all except USP18 being direct targets of ISGylation [95,105,106,107,108,109]. ISG15 itself stabilizes USP18 to turn off IFN-I signaling as a type of negative feedback regulation. Here, non-covalent, hydrophobic interactions between free ISG15 and USP18 mask the ubiquitination sites on USP18, preventing it from being degraded by the proteasome [95]. Stabilized USP18 then initiates a feed-forward mechanism, cleaving ISG15 from proteins to successfully dampen IFN-I signaling [95]. Intriguingly, ISGylation of TRIM25 can both activate and inhibit its activity, with autoISGylation specifically blocking its function as an ISG15 E3 ligase, but this was demonstrated for only one substrate [110]. These ISG15-dependent mechanisms are shown in Figure 2.

### 4.3. Viruses Have Evolved Mechanisms That Antagonize ISG15-Dependent Regulation of Cytosolic RNA and DNA Sensing Pathways to Dampen IFN-I Signaling

Since ISGylation of cytosolic RNA and DNA sensor pathway proteins generally amplifies IFN-I signaling, many viruses have evolved mechanisms to thwart ISGylation to evade immune system detection. These mechanisms control the levels of ISGylated proteins either indirectly by targeting different steps of the ISGylation pathway, such as the ISGylation machinery (E1/E2/E3 ubiquitin proteins), or directly through deISGylation. Here, we focus on direct and indirect viral mechanisms that interfere with the ISGylation of cytosolic RNA and DNA sensors and adaptors and other downstream components of the IFN-I pathway. We define direct effects as deISGylation or sterically blocking ISGylation or ISG15-protein interactions. These viral mechanisms are summarized in Table 2.

A study performed by Zhu and colleagues revealed a novel mechanism whereby porcine respiratory syndrome virus (PRRSV) non-structural protein 5 (NSP5) hijacked UBCH8/UBE2L6 to decrease IFN-I signaling. Here, the authors showed that transfection of Marc-145 cells with increasing concentrations of a plasmid encoding NSP5 increased expression of UBCH8/UBE2L6 but reduced expression of RIG-I, MDA5, and total ISGylation in a dose-dependent manner [111]. Immunoprecipitation of MDA5 or RIG-I in Marc-145 cells transfected with NSP5 revealed increased K43-linked ubiquitination for both proteins [111], suggesting that UBCH8/UBE2L6 promoted the degradation of MDA5 and RIG-I through the proteasomal pathway. Not surprisingly, cells with silenced UBCH8/UBE2L6 had reduced PRRSV viral titers after infection compared to control cells [111]. This work revealed a mechanism whereby NSP5 exploits host UBCH8/UBE2L6 to enhance its function as a ubiquitin-conjugating enzyme, leading to the degradation of MDA5 and RIG-I and a corresponding reduction in total cellular ISGylation.

ISGylation of MDA5 promotes its oligomerization and subsequent activation of MAVS and downstream IFN-I signaling. The SARS-CoV-2 non-structural protein 3 (NSP3), which encodes a PLpro, deISGylated MDA5, as HEK293T cells that were transfected with a catalytically inactive mutant of PLpro had increased levels of ISGylated MDA5 compared to the wild-type PLpro-transfected cells [82]. This deISGylation activity was important in suppressing IFN-I signaling, as HeLa cells transfected with the catalytically inactive PLpro and infected with EMCV had elevated transcript levels of *IFNB1*, *CCL5*, and *IFIT1* compared to wild-type transfected cells [82]. Similar results were observed for SARS-CoV-2 viral protein NSP7. Transfection of an immortalized, non-tumorigenic human bronchial epithelial cell line (BEAS-2B) with an empty vector or a vector encoding NSP7 resulted in lower levels of ISGylated MDA5 in NSP7-transfected cells after treatment with poly(I:C) [112]. In addition to SARS-CoV2, African swine fever virus (ASFV) was demonstrated to antagonize the ISGylation of MDA5 via the MGF_360-4L protein. PAMs that were infected with wild-type ASFV or a mutant with the gene encoding MGF_360-4L deleted (ASFV-ΔMGF_360-4L) displayed lower levels of ISGylated MDA5 in the wild-type virus infected cells [113]. Moreover, domestic pigs infected with ASFV-ΔMGF_360-4L exhibited markedly milder clinical symptoms and increased survivability compared to those infected with the wild-type virus [113]. These results highlighted MGF_360-4L as a key virulence factor that suppressed host immunity by modulating MDA5 ISGylation.

Regulation of RIG-I activity by ISG15 is complex (Figure 2), and not much work has been done to elucidate the viral countermeasures against the different ISG15-mediated pathways that control the activation or degradation of RIG-I. One of the ways in which ISG15 non-covalently regulates RIG-I is through the facilitation of LRRC25-mediated autophagic degradation of RIG-I to turn off IFN-I signaling. Research performed by Yang and colleagues revealed that FMDV non-structural protein 3A (NSP3A), as well as other picornaviruses (listed in Table 2), increased protein abundance of LRRC25 in HEK293T cells that were transfected with a plasmid encoding this viral protein [114]. Cells that were transfected with LRRC25 had lower levels of RIG-I, and this effect was dose-dependent [114]. In this study, NSP3A was shown to directly interact with LRRC25 [114], so perhaps this interaction facilitates binding between LRRC25 and ISG15, although this hypothesis requires further exploration.

While there is currently no evidence that the RNA sensing pathway adaptor MAVS is directly regulated by ISGylation or non-covalent interactions with ISG15, viral countermeasures exist to indirectly control global ISGylation through MAVS. Studies of HCV revealed that the viral protease nonstructural protein 3/nonstructural protein 4A (NS3/4A) cleaved MAVS, blocking its interaction with RIG-I and lowering the activity levels of the *IFN-β* promoter in a human hepatoma cell line (Huh7) and a modified osteosarcoma cell line (UNS3/4A) [115]. In liver samples from patients chronically infected with HCV, increased abundance of the cleaved form of MAVS, which lacks the ability to interact with RIG-I, corresponded with decreased levels of total ISGylated proteins [115].

Regarding the cGAS-STING-mediated DNA sensing pathway, a membrane fusion machinery protein (OPG147) belonging to the monkeypox virus (MPXV) blocked ISGylation and oligomerization of STING by directly binding to K289 on STING in HEK293 cells, THP-1 cells, and HeLa cells [116]. This binding resulted in lower transcriptional expression of the pro-inflammatory factors IFNB1, CXCL10, and IL16 and elevated vaccinia virus (VACV) titers in infected THP-1 cells [116]. More work is required to uncover the viral mechanisms that counteract the ISG15-mediated control of the STING-activated DNA sensing pathways.

ISGylation of TRIM25 positively and negatively regulates the function of this protein to control the IFN-I response, with ISGylated TRIM25 generally functioning to activate IFN-I signaling. One study showed that transfection of HEK293T cells with PRRSV NSP1α decreased the level of ISGylated TRIM25 in these cells [117]. Destabilization of ISGylated TRIM25 appeared to be mediated by the proteasome and not by the PLpro activity of NSP1α, as transfection of PAM-KNU cells with a catalytically inactive form of NSP1α still resulted in lower levels of TRIM25, while chemical perturbation of the proteasome with MG132 restored the levels of TRIM25 in HEK293T cells [117].

Taken together, these findings highlight the diversity in viral countermeasures against ISG15-mediated regulation of cytosolic RNA and DNA sensor pathways to control IFN-I signaling and provide insight into potential therapeutic targets that might function by blocking these viral countermeasures, such as viral protease inhibitors.

**Table 2:** Summary of viral countermeasures that affect ISG15-mediated regulation of cytosolic DNA and RNA sensing pathways.

Substrate	Mechanism of Action	Cell/Model System	Virus	Viral Protein	Functional Outcome	Literature
MDA5	DeISGylates MDA5, hijacks UBCH8/UBE2L6	HEK293T, NHLFs, A549-hACE2, HeLa, BEAS-2B, PAMs, Marc-145, domestic pigs	SARS-CoV-2, ASFV, PRRSV	NSP3 (PLpro), NSP7, MGF_360_4L, NSP5	Destabilization of MDA5, lower total ISGylation, reduced IFN-I signaling, elevated clinical symptoms and mortality	[82,111,113]
RIG-I	No known direct mechanisms, stabilizes LRRC25, hijacks UBCH8/UBE2L6	HEK293T, PAMs, Marc-145	FMDV, SVV, EV71, EMCV, PRRSV	3A, NSP5	Destabilization of RIG-I, lower total ISGylation, higher viral titers in infected host cells	[111,114]
MAVS	No known direct mechanisms; cleaves MAVS	Huh7, Huh7-HP, UNS3/4A, human liver cells from patients with chronic HCV infection	HCV	NS3/4A	Lower total ISGylation, decreased *IFN-β* promoter activity	[115]
cGAS	No known direct mechanisms; inhibition of cGAS-mediated signaling	HEK293, HeLa, THP-1, bronchoalveolar lavage from mice	MPXV, VACV	OPG147	Reduced IFN-I signaling	[116]
STING	Physically blocks ISGylation sites on STING	HEK293, HeLa, THP-1, bronchoalveolar lavage from mice	MPXV, VACV	OPG147	Inhibition of oligomerization, reduced IFN-I signaling	[116]
TRIM25	No known direct mechanisms; destabilizes ISGylated TRIM25	HEK293T, PAM-KNU, Marc-145, PK-15	PRRSV	NSP1α	Proteasome-mediated degradation of TRIM25, reduced IFN-I production, increased viral DNA copies in host cells	[117]

## 5. The Effect of ISG15 on IFN-I Signaling Exhibits Species-Specific Differences

The enzymatic ISGylation cascade and its downstream modulation of cytosolic RNA and DNA sensing pathways are functionally conserved across vertebrates [68,82,85,88,89,91,92,94,99,103,118], even though the enzymes catalyzing the ISGylation of protein substrates may be species-dependent [119]. However, some sufficient differences have been identified between mice and humans, the most extensively studied organisms in this context, regarding phenotypic consequences of *ISG15* deficiency.

In mice, *ISG15* deficiency resulted in increased susceptibility to viral infections, such as influenza A/WSN/33 and influenza B/Lee/40 [118]. These mice also showed increased susceptibility to infections caused by HSV-1, murine gammaherpesvirus 68, and Sindbis virus [118]. The authors of this study suggested that this outcome is likely attributable to the lack of ISGylation of antiviral proteins and therefore lower activity of the IFN-I response.

In contrast, in humans, inherited *ISG15* deficiency is not associated with cellular susceptibility to any viruses, consistent with the absence of viral diseases in these patients, but leads to severe interferonopathy and autoinflammation [120]. At the same time, impaired ISG15 secretion by granulocytes in response to mycobacterial stimulation resulted in reduced IFN-γ production by lymphocytes including NK cells, a factor likely underlying the increased susceptibility to mycobacterial infections [120]. This effect appears to be linked to the cytokine-like activity of secreted ISG15. Subsequently, the same group demonstrated that ISG15-deficient patients display unanticipated cellular, immunological, and clinical signs of enhanced IFN-α/β immunity, like the inherited autoinflammatory interferonopathies Aicardi–Goutières syndrome and spondyloenchondrodysplasia. Mechanistically, they revealed that the loss of intracellular ISG15 prevents the stabilization and accumulation of USP18, thereby leading to sustained and amplified IFN-I responses [93]. Speer and colleagues demonstrated that fibroblasts derived from patients with *ISG15* deficiency exhibited enhanced antiviral protection, and that complementary ISG15 expression reduced this level of viral resistance to that observed in wild-type control cells [94]. The species-specific enhancement of antiviral immunity observed in ISG15-deficient humans (a “gain-of-function” effect) was linked to the lack of USP18 stabilization by ISG15 and, consequently, to the disruption of the negative feedback mechanism regulating the IFN-I signaling pathway in humans [94].

## 6. Summary

**Figure 3:**
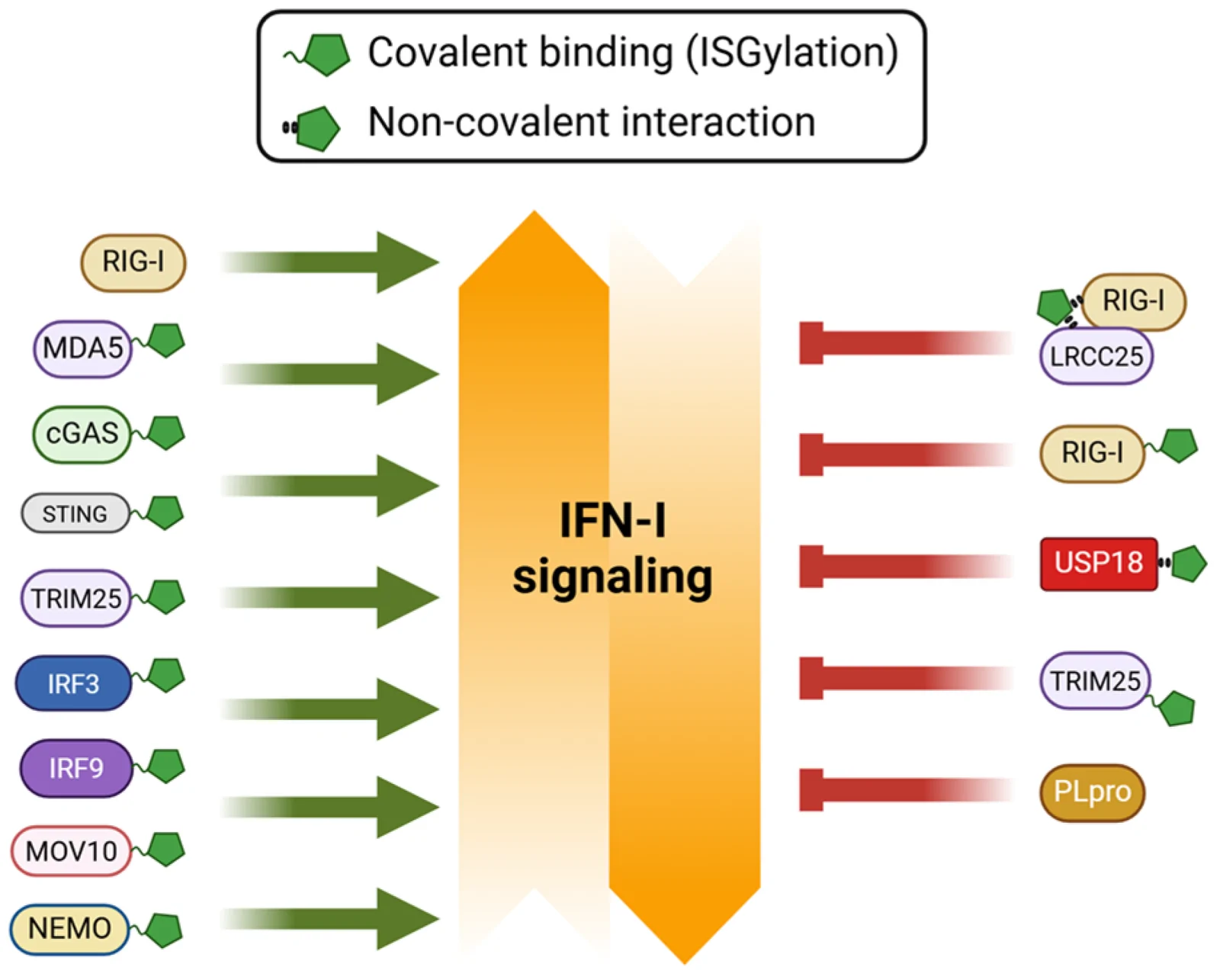
Bidirectional regulation of type I interferon (IFN-I) signaling by interferon-stimulated gene 15 (ISG15). ISG15, a small ubiquitin-like modifier, covalently or non-covalently interacts with components of the cytosolic DNA and RNA sensing pathways to amplify or inhibit IFN-I signaling. ISG15 covalently binds to the cytosolic sensors melanoma differentiation-associated protein 5 (MDA5), retinoic acid-inducible gene I (RIG-I), and cyclic GMP-AMP synthase (cGAS) and adaptor stimulator of interferon genes (STING) within nucleic acid sensing pathways, enhancing or repressing IFN-I signaling. Simultaneously, ISGylation of interferon response factor 3 (IRF3), interferon response factor 9 (IRF9), Moloney leukemia virus 10 (MOV10), and NF-κB essential modulator (NEMO) promotes the robust transcriptional activation of downstream ISGs and NF-κB-activated genes. Non-covalent binding between ISG15 and ubiquitin-specific peptidase 18 (USP18), a negative regulator of IFN-I signaling, stabilizes USP18. Covalent binding of ISG15 to tripartite motif-containing protein 25 (TRIM25) activates or, in the case of auto-ISGylation, inhibits IFN-I signaling. Non-covalent ISG15-mediated interactions between leucine-rich repeat-containing protein 25 (LRRC25) and RIG-I lead to autophagic degradation of RIG-I and therefore decreases IFN-I signaling. Viral countermeasures such as papain-like proteases (PLpro) dampen IFN-I signaling to evade the host immune response. Activating effects are indicated by green arrows; inhibitory effects, by red lines; non-covalent ISG15 binding is shown by dotted black lines. Created in BioRender. White, M. and Iordanskiy, S. (2026) https://BioRender.com/t25pdl8.

This review highlights the dual regulatory roles of ISG15 in controlling the balance between amplifying IFN-I signaling and shutting it down. The major activities of ISG15 in the regulation of IFN-I signaling are illustrated in Figure 3. On one hand, ISG15 acts as a potent pro-inflammatory and antiviral agent: through covalent conjugation (i.e., ISGylation), it tags cytosolic nucleic acid sensors, downstream adaptors, and viral proteins to fuel IFN-I expression and halt viral replication. On the other hand, it functions as a negative feedback regulator by binding non-covalently to USP18, which stabilizes this interferon-inducible factor so it can shut down signaling via the IFNAR2 subunit through a non-catalytic scaffolding mechanism. Simultaneously, USP18 utilizes its canonical protease activity to deISGylate cytosolic nucleic acid sensors like MDA5 and cGAS and the adaptor STING to resolve inflammation triggered by pathogens or tissue injury [121]. The ultimate biological effect of ISG15 is dictated by a multitude of variables: the specific substrate, modifying enzyme, cell or model system, viral stimulus or DAMP, and the dynamics of the antiviral response.

While over the past two decades these ISG15-dependent mechanisms have been characterized in detail, important questions remain regarding their temporal coordination. A common feature appears to be that early in infection, ISGylation rapidly amplifies IFN-I signaling to clear pathogens; later, negative feedback loops terminate this response once the threat is resolved. Deciphering the temporal functional transition of ISG15 is vital and warrants further study, as a disruption in the processes that dampen the inflammatory response can prevent inflammation from resolving and cause it to progress to a chronic form. This dynamic is especially critical in pathogen-unrelated contexts, such as cancer-associated inflammation, tissue damage, fibrosis, autoimmunity, and likely inflammaging, where persistent IFN-I signaling drives long-term pathology in the absence of viral infection. Indeed, recent work from our group and others demonstrated that ionizing radiation, a potent inducer of DNA damage and DAMPs, triggered the prolonged expression of retroelements [46,48,50,122,123,124]. This sustained retroelement activity can lead to recurrent IFN-I activation, ultimately overwhelming negative feedback mechanisms like the stabilization of USP18 by ISG15.

Recent studies on the ISG15-dependent regulation of antiviral and inflammatory responses through positive and negative feedback loops within the IFN-I system raise the question of how these mechanisms might be harnessed to combat viral infections and mitigate tissue damage-induced inflammation. This is a plausible but risky approach: while enhancing ISGylation can bolster antiviral sensing, it carries a risk of exacerbating interferonopathies, fibrosis, or autoimmunity depending on the physiological context. Conversely, targeting the immunosuppressive functions of USP18, specifically through its interaction with ISG15, could improve cancer immunotherapy outcomes by overcoming targeted therapy resistance and managing recalcitrant infections [121]. Over the past decade, several small molecules targeting USP18 have emerged. While compounds like β-lapachone and hyperoside directly inhibit USP18’s enzymatic activity [125,126], novel next-generation inhibitors, such as ZHAWOC8655, have advanced the field by disrupting intracellular protein–protein interaction between USP18 and ISG15 and reducing USP18’s protease activity [127]. This highly specific disruption elevates global cellular ISGylation, triggering robust apoptosis and potent antineoplastic responses in lung cancer models.

A deeper understanding of how ISG15 modulates IFN-I signaling, combined with how IFN-I regulates ISG15 expression and downstream ISGylation, is essential for uncovering the fine-tuned mechanisms that govern immune responses and inflammation as well as the resolution of these processes. Ultimately, these insights will guide the design of targeted therapies for viral infections, malignancies, and chronic inflammatory disorders.

## 7. Concise Limitations

Several limitations should be considered when interpreting the findings of this review. First, while we conducted a comprehensive search using PubMed as the primary database, our selection was restricted to English-language, peer-reviewed publications, which may exclude relevant findings from non-English sources. Second, significant heterogeneity in experimental methodologies, cell types, and animal models across the cited studies complicates direct, quantitative comparisons of ISG15 function and IFN-I pathway regulation. Finally, because the field of IFN-I signaling is rapidly evolving, ongoing functional characterizations may alter current understandings of the cytosolic PRR-ISG15 regulatory axis faster than published literature reflects.

## Figures and Tables

**Table 1:** Summary of several of the RNA- and DNA-sensing pathway receptors and downstream proteins and their regulation by ISG15.

Substrate	Mechanism of Action	Modification Site	Modifying Enzyme	Cell/Model System	Virus/Stimulus	Functional Outcome	Literature
MDA5	ISGylation	K23, K43, K983	UBA7/ UBE1L, UBCH8/ UBE2L6, HERC5/ HERC6	HEK293, HEK293T, MEFs, HeLA NHLFs, SVGAs, A549-hACE2, mouse liver	HMW-Poly(I:C), SeV, DENV, ZIKV, SARS-CoV-2, *L. monocytogenes*, ZIKV, EMCV-RNA, IAV, RABV-Le, West Nile virus, IFN-β	Oligomerization, activation of IFN-I signaling	[80,82]
RIG-I	ISGylation, non-covalent binding, indirect regulation through sequestering UBCH8	K257, K456	UBA7/ UBE1L, UBCH8/ UBCM8/ UBE2L6	HeLa, HEK293T, HepG2, RAW264.7, COS-7, iBMMs, FTE-194, mouse liver	SeV, LPS, IFN-β, PR8, MG132, IFN-α2a, poly(I:C), *L. monocytogenes*	Stabilization, autophagic degradation, proteasomal degradation, inhibition or activation of IFN-I signaling	[76,80,83,84,85,86]
cGAS	ISGylation	K21, K187, K219, K458	UBA7/ UBE1L, UBCH8/ UBE2L6, HERC5, ARIH1	HEK293 HEK293T, HAECs, L929, HFF, HeLa, AH109, MEsF	IFN-β, HT-DNA, HSV-1	Oligomerization, activation of ISRE and NF-κB promoters	[87,88]
STING	ISGylation	K149, K150, K166, K203, K224, K221, K229, K236, K238, K276, K289, K325 K347, K338, K370	UBA7/ UBE1L, UBCH8/ UBE2L6, HERC5/ HERC6	HEK293A, THP-1, ZFINs, HEK293T, mouse peritoneal macrophage	HIV-1, SR-717, B-DNA	Oligomerization, activation of IFN-I response	[89,90,91]
USP18	Non-covalent binding	None identified	None identified	MEFs, HEK293T, U3A, HLLR1-1.4, A549, mouse bone marrow, mouse neurons, *E. coli*	IFN-β, VSV, IFN-α	DeISGylation, inhibition of JAK1 binding to IFNAR2, downregulation of IFN-I signaling	[92,93,94,95,96,97,98]

## Data Availability

No new data were created or analyzed in this study. Data sharing is not applicable to this article.

## Abbreviations

The following abbreviations are used in this manuscript:

ADAP	Adhesion and degranulation-promoting adapter protein
AIM2	Absent in melanoma 2
ARIH1	Ariadne RBR E3 ubiquitin protein ligase 1
ASFV	African swine fever virus
Aza	5-azacytidine
BMM	Bone marrow-derived macrophage
CCHFV	Crimean–Congo hemorrhagic fever virus
cGAS	Cyclic GMP-AMP synthase
CMV	Cytomegalovirus
DAMP	Damage-associated molecular pattern
DDX41	DEAD-box helicase 41
DSB	Double-stranded break
dsDNA	Double-stranded DNA
dsRNA	Double-stranded RNA
DUB	Deubiquitinating enzyme
EMCV	Encephalomyocarditis virus
ER	Endoplasmic reticulum
ERV	Endogenous retrovirus
ESCRT	Endosomal sorting complex required for transport
FMDV	Foot-and-mouth disease virus
HDAC6	Histone deacetylase 6
HEK293	Human embryonic kidney cell line 293
HepG2	Hepatic glycoprotein 2/hepatocellular carcinoma cell line 2
HERC5	HECT and RLD domain-containing E3 ubiquitin protein ligase 5
Herc6	HECT and RLD domain-containing E3 ubiquitin protein ligase 6
HIV-1	Human immunodeficiency virus type 1
HSV-1	Herpes simplex virus type 1
IAV	Influenza A virus
IBV	Influenza B virus
iBMM	Immortalized bone marrow-derived macrophage
IFI16	Interferon gamma-inducible protein 16
IFIT1	Interferon-induced protein with tetratricopeptide repeats 1
IFN	Interferon
IFN-I	Type I interferon
IFN-II	Type II interferon
IFN-α	Interferon-alpha
IFN-β	Interferon-beta
IFN-γ	Interferon-gamma
IFN-ε	Interferon-epsilon
IFN-κ	Interferon-kappa
IFN-ω	Interferon-omega
IFNAR	Interferon-alpha/beta receptor
IFNAR1/2	Interferon-alpha/beta receptor subunits 1 and 2
IKKε	IκB kinase-epsilon
IL-6/IL-10/IL-12/IL-18	Interleukin 6/10/12/18
IL-18Rα	Interleukin 18 receptor alpha
IP	Immunoprecipitation
IPS-1	Interferon promoter-stimulator 1
IRF3/IRF7/IRF9	Interferon regulatory factor 3/7/9
ISG	Interferon-stimulated gene
ISG15	Interferon-stimulated gene 15
ISGF3	Interferon-stimulated gene factor 3
ISRE	Interferon-stimulated response element
JAK1	Janus kinase 1
LFA-1	Lymphocyte function-associated antigen 1
LGP2	Laboratory of genetics and physiology 2
LRRC25	Leucine-rich repeat containing protein 25
MAVS	Mitochondrial antiviral-signaling protein
MDA5	Melanoma differentiation-associated protein 5
MEF	Mouse embryonic fibroblast
MERS-CoV	Middle East respiratory syndrome coronavirus
MPXV	Monkeypox virus
MS	Mass spectrometry
MX1/MxA	Myxovirus resistance 1/Myxovirus resistance protein A
MOV10	Moloney leukemia virus 10
Nbs1	Nijmegen breakage syndrome 1
NEMO	NF-κB essential modulator
NF-κB	Nuclear factor kappa-light-chain-enhancer of activated B cells
NK	Natural killer
NLRP3	NLR family pyrin domain-containing 3
NSP	Non-structural protein
OTU	Ovarian tumor
PAM	Porcine alveolar macrophage
PAMP	Pathogen-associated molecular pattern
PLpro	Papain-like protease
poly(I:C)	Polyinosinic–polycytidilic acid
PRR	Pattern recognition receptor
PRRSV	Porcine reproductive and respiratory syndrome virus
PTM	Post-translational modification
RBR	RING-between-RING
RIG-I	Retinoic acid-inducible gene I
RING	Really interesting new gene
RLR	RIG-I-like receptor
RNF125	RING finger protein 125
SARS-CoV-2	Severe acute respiratory syndrome coronavirus 2
SeV	Sendai virus
siRNA	Small interfering RNA
ssDNA	Single-stranded DNA
ssRNA	Single-stranded RNA
STAT1/STAT2	Signal transducer and activator of transcription 1/2
STING	Stimulator of interferon genes
TBK1	TANK-binding kinase 1
TF	Transcription factor
TNF-α	Tumor necrosis factor alpha
TRIM25	Tripartite motif-containing protein 25
UBA7	Ubiquitin-like modifier-activating enzyme 7
UbcH8	Ubiquitin-conjugating enzyme human 8
UbcM8	Ubiquitin-conjugating enzyme mouse 8
UBE1L	Ubiquitin-activating enzyme E1-like
UBE2L6	Ubiquitin-conjugating enzyme E2-like 6
UBL	Ubiquitin-like protein
USP18/USP24	Ubiquitin-specific peptidase 18/24
VACV	Vaccinia virus
